# Cellular Mechanisms and Therapies in Wound Healing: Looking toward the Future

**DOI:** 10.3390/biomedicines9111611

**Published:** 2021-11-04

**Authors:** Stefano Bacci

**Affiliations:** Department of Biology, Research Unit of Histology and Embriology, University of Florence, Viale Pieraccini 6, 50139 Florence, Italy; stefano.bacci@unifi.it

## 1. Original Research Articles: Significant Progress on Various Fronts

The high professionalism of these publications consists, on the one hand, of revealing some of the mechanisms underlying wound healing and, on the other hand, of proposing alternative therapies for the fine control of inflammation following injury to avoid fibrotic scars or impaired wounds.

## 2. Cellular Mechanisms in Wound Healing

Wound healing is the process that makes organisms resilient to injuries, enabling survival. It is of fundamental importance for life, and its basic pathways have been conserved throughout evolution. Wound healing is divided into three phases: inflammation, proliferation, and remodeling, whose mechanisms partially overlap both spatially and temporally. After an injury, clotting occurs due to immediate interactions among endothelial cells and platelets and activation of the coagulation cascade. Mediators released during this early process trigger an inflammatory reaction summoning neutrophils and macrophages from the bloodstream. In turn, these cells produce proinflammatory cytokines and growth factors, resulting in the recruitment of stromal cells and their differentiation into myofibroblasts, which are responsible for wound contraction and extracellular matrix deposition. These cells are able to stimulate endothelial and epithelial cell proliferation in the wound site to induce neoangiogenesis and re-epithelialization, respectively. Clot and tissue debris are eventually removed by macrophages and extracellular hydrolases (matrix metalloproteases, elastase, and plasmin), and tissue repair proceeds toward and terminates with scarring [1,2].

## 3. Photobiomodulation in Effector Cells of Wound Healing

Photobiomodulation is defined as a form of light therapy that utilizes non-ionizing light sources, including lasers, light-emitting diodes, and/or broadband light, in the visible (400–700 nm) and near-infrared (700–1100 nm) electromagnetic spectrum. It is a nonthermal process involving endogenous chromophores eliciting photophysical (i.e., linear and nonlinear) and photochemical events at various biological scales. This process results in beneficial therapeutic outcomes including, but not limited to, the alleviation of pain, immunomodulation, and promotion of wound healing, and tissue regeneration [3].

Genah et al. studied the cellular and molecular events associated with anti-inflammatory activity in wound healing and proposed a photobiomodulation therapy using a dual-wavelength NIR laser source. In this context, human dermal fibroblasts were exposed to a mix of inflammatory cytokines, such as IL-1 and TNF-alpha, followed by laser treatment. Molecules, such as iNOS and the COX-2/mPGES-1/PGE2 cascade, were upregulated by the cytokine mix, while therapy reverted their levels and activities. The same behavior was observed with the endothelial growth factor, involved in neovascularization as well as NF-kB. In terms of morphology, differences in expression and distribution of cytoskeletal proteins were observed following treatments. In fact, tubulin, F-actin, and smooth muscle actin changed their organization upon cytokine stimulation, while therapy re-established the basal localization. Similar changes were observed for collagen I and Matrix metalloproteinase-1.

For the authors, this study demonstrates that NIR laser therapy is effective in controlling fibroblast activation induced by IL-1 and TNF-alpha (for more details about this article, see [4]).

Rossi et al. presented a study on the effects of blue LED light on the proliferation and metabolism of human fibroblasts from healthy skin cocultured with keratinocytes. Different light doses were used to treat the cells, evincing inhibitory and stimulatory effects following a biphasic dose behavior. Electrophysiology was used to investigate the effects on membrane currents and showed no significant differences between cells. Raman spectroscopy revealed the mitochondrial cytochrome C oxidase dependence on blue light irradiation; a significant decrease in the peak intensity of the healthy fibroblast was evinced, while it was less pronounced in keratinocytes. The authors proposed that the blue LED light could be used to modulate the metabolism and proliferation of human fibroblasts, and the effects on wound healing were evident when studying the fibroblast and keratinocyte cocultures (for more details about this article, see [5]).

## 4. Proposed Therapies for Chronic wounds

The definition of a chronic wound is a wound that does not heal in an orderly set of stages and in a predictable amount of time. From a practical point of view, wounds that do not heal within 6 to 8 weeks are most often considered chronic. Chronic venous ulcers are associated with an extremely high psychosocial burden in terms of morbidity, loss of productivity, functional disability, and emotional distress, causing depression and social isolation. A recent retrospective analysis of medical care beneficiaries identified that ~8.2 million people had wounds with or without infections. The treatment of chronic wounds is an area of intense study since each chronic wound is unique, with a unique set of physiological and social circumstances preventing or slowing wound healing [6].

Bayer et al. investigated the effects of Vivostat Platelet-Rich Fibrin^®^ (PRF), an autologous platelet concentrate used for the local treatment of chronic or complicated wounds. The results showed that 77.1% of the treated patients benefited from Vivostat PRF^®^ therapy. Additionally, on other subjects, in generated bilateral gluteal wounds, they analyzed the influence of the treatment on the expression of two genes, in keratinocytes, which are induced during wound healing as human beta Defensin-2 (hBD-2) and human beta-Defensin-3 (hBD-3). This analysis revealed that only 70% of the individuals showed a relevant hBD-2 and hBD-3 gene induction after therapy. The authors concluded that not all human individuals are sensitive to wound therapy with autologous thrombocyte products because they lack specific extra- or intracellular receptor and/or signal transduction molecules (for more details about this article, see [7]).

## 5. Cellular Mechanisms, Epigenetic Modification, and Proposed Therapies in Keloids

A keloid is an abnormal proliferation of scar tissue that forms at the site of cutaneous injury (e.g., on the site of a surgical incision or trauma); it does not regress and grows beyond the original margins of the scar. They occur where trauma, surgery, blisters, vaccinations, acne, or body piercing have injured the skin. Less commonly, keloids may form in places where the skin has not had a visible injury. Keloids differ from normal mature scars in composition and size. Some people are prone to keloid formation and may develop them in several places. Keloids are more common in African Americans. They are seen most commonly on the shoulders, upper back, and chest, but they can occur anywhere. When a keloid is associated with a skin incision or injury, the keloid scar tissue continues to grow for a time after the original wound has closed, becoming larger and more visible until it reaches a final size. They generally occur in people between 10 and 30 years of age and affect both sexes equally, although they may be more common among young women with pierced ears. Keloids may form over the breastbone in people who have had open-heart surgery. There are several treatments available, but none have been shown to be more effective than others [8].

Alghamdi et al. investigated the role of keloid keratinocytes in regulating collagen production by primary fibroblasts in vitro. Keloid cells were obtained from removed patient tissue, whereas normal skin cells were from elective surgery procedures. Fibroblasts and keratinocytes were isolated, cultured, and a particular coculture system was used to investigate the effects of keratinocytes on collagen production. Keloid fibroblasts produced significantly more collagen than normal skin fibroblasts. When keloid keratinocytes were added to normal skin fibroblasts, expression of collagen was significantly upregulated in most samples, but when added to keloid fibroblasts, collagen I production was significantly reduced. Interestingly, keloid keratinocytes appear to decrease collagen production by keloid fibroblasts. The authors suggested that signaling in both keratinocytes and fibroblasts is disrupted in keloid pathology (for more details about this article, see [9]).

Alghamdi et al. investigated the hypothesis that keloidogenesis may be driven by epigenetic changes, particularly, DNA methylation. DNA methylation is known to occur commonly at CpG islands and was traditionally linked to transcriptional silencing. These CpG islands are found in high densities in the promoter regions of genes, where transcription of DNA begins, and thus regulate gene transcription. This study explored whether altered collagen deposition by keloid fibroblasts could at least in part be explained by epigenetic changes sustainably and heritably altering the gene expression profile in these cells. The results showed significant DNA methylation changes at the CpG sites in multiple genes in fibroblasts of keloid scars compared to control skin fibroblasts.

The CpG sites were associated with genes such as tankyrase 2 (TNKS2), family with sequence similarity 45 members (FAM45B), LOC723972, growth arrest-specific 7 (GAS7), rhomboid domain containing 2 (RHBDD2), and calcium/calmodulin-dependent protein kinase kinase 1 (CAMKK1), and with many cellular functions other than the most common regulators of the signaling network analysis. The authors proposed that the methylation status of keloids could be implicated in the mechanisms of keloid scar formation and remission (for more details about this article, see [10]).

Magni et al. analyzed the effects of blue LED light irradiation on human fibroblasts, isolated from both keloids and perilesional tissues. Biochemical assays and specific staining were used to assess cell metabolism, proliferation, and viability. Micro-Raman spectroscopy was used to explore the direct effects of blue LED light on the cytochrome C oxidase and by patch-clamp recording, the effects of the irradiation on ionic membrane currents. The results showed that blue LED light can modulate cell metabolism and proliferation, with a dose-dependent behavior, and that these effects persist at least 48 h after treatment. Furthermore, the highest doses of irradiation reduce cell viability 24 h after the treatment in keloid-derived fibroblasts and 48 h in perilesional fibroblasts. Electrophysiological recordings showed that a medium dose of blue LED light induced an enhancement of voltage-dependent outward currents elicited by a depolarizing ramp protocol. The authors concluded that blue light irradiation can be considered an innovative and minimally invasive approach in the management of hypertrophic scars and keloids (for more details about this article, see [11]).

## 6. Conclusions

At the conclusion of this editorial, I would like to reiterate the concept that *a deep knowledge of cellular infiltrate responses remains fundamental to the study of wound healing. In fact, there must be an awareness of the fact that the modulation of effector cells (fibroblasts or myofibroblasts) is related to other cell types deeply involved in wound healing (for example, mast cells or dendritic cells) that are present in the skin microenvironment.* Bearing this in mind, it is hoped that various therapies will be proposed and improved in the future.
